# Venezuelan Equine Encephalitis Virus Transmission and Effect on Pathogenesis

**DOI:** 10.3201/eid1208.050841

**Published:** 2006-08

**Authors:** Darci R. Smith, Patricia V. Aguilar, Lark L. Coffey, Gregory D. Gromowski, Eryu Wang, Scott C. Weaver

**Affiliations:** * University of Texas Medical Branch, Galveston, Texas, USA

**Keywords:** Venezuelan equine encephalitis virus, arbovirus, alphavirus, mosquito, saliva, transmission, research

## Abstract

Quantifying the dose of an arbovirus transmitted by mosquitoes is essential for designing pathogenesis studies simulating natural infection of vertebrates. Titration of saliva collected in vitro from infected mosquitoes may not accurately estimate titers transmitted during blood feeding, and infection by needle injection may affect vertebrate pathogenesis. We compared the amount of Venezuelan equine encephalitis virus collected from the saliva of *Aedes taeniorhynchus* to the amount injected into a mouse during blood feeding. Less virus was transmitted by mosquitoes in vivo (geometric mean 11 PFU) than was found for comparable times of salivation in vitro (mean saliva titer 74 PFU). We also observed slightly lower early and late viremia titers in mice that were needle injected with 8 PFU, which represents the low end of the in vivo transmission range. No differences in survival were detected, regardless of the dose or infection route.

Designing pathogenesis studies for arboviruses that accurately simulate natural infection requires quantifying the amount of virus transmitted. Virus assays on mosquito saliva can be used to estimate the amount transmitted to vertebrates during blood feeding. However, amount of virus collected in vitro may not accurately reflect mosquito transmission.

Indirect and direct methods can be used to quantify virus delivered in mosquito saliva. Indirect methods include comparing times of death of animals exposed to a mosquito bite with those infected parenterally with known doses and comparing the time between mosquito feeding and development of viremia with the time between needle injection and development of the same viremia level. Direct methods include quantifying virus salivated into drops of blood, virus detected in vertebrate tissues immediately after mosquito feeding, virus salivated into blood agar, and virus salivated into fluids such as oil. Saliva collection in oil-filled capillary tubes, first described by Hurlbut (*1*), is widely used. Chamberlain et al. (*2*) compared several indirect and direct methods for quantifying arbovirus transmission and concluded that allowing mosquitoes to feed on serum (similar to the capillary method) is less efficient in detecting virus than other methods. Since most saliva is expectorated during probing, salivation into hanging drops or capillary tubes may be inaccurate because mosquitoes do not need to salivate to locate a blood vessel.

The amount of several arboviruses transmitted by mosquitoes has been estimated by using artificial saliva collection (*1**–**12*). Up to 3 log_10_ PFU of eastern equine encephalitis virus is deposited into capillary tubes filled with oil by the vector *Culiseta melanura* (*9*). Capillary collection and real-time reverse transcription (RT)–PCR estimate that *Culex pipiens pipiens* saliva contains an average of 4.3 log_10_ PFU of West Nile virus (WNV), with a range of 0.5 to 5.3 log_10_ (*8*). Recently, we estimated that the epidemic Venezuelan equine encephalitis virus (VEEV) vector, *Aedes* (*Ochlerotatus*) *taeniorhynchus,* salivates 0.2–3.2 log_10_ PFU into oil-filled capillary tubes (*12*).

Vector saliva enhances infection with many pathogens (*13**–**18*), and mosquito saliva is reported to enhance infection by some arboviruses. Deer and chipmunks infected with La Crosse virus by the bite of *Ae*. (*Och*.) *triseriatus* have higher and longer viremias than animals infected by needle (*19*). Mice exposed to uninfected mosquitoes and injected at the feeding site with Cache Valley virus develop enhanced viremia and seroconversion compared with unbitten mice or to those co-injected with virus and mosquito saliva (*20*). Mice have higher seroconversion rates to vesicular stomatitis virus when infected by *Ae*. *triseriatus* than by needle injection (*21*). Cytokine expression in the skin of mice infected with Sindbis virus differs after injection with mosquito salivary gland extracts than after injection with virus alone (*22*).

Other studies report no enhancement of arbovirus infection by vector saliva or feeding. Hamsters infected with WNV by mosquitoes or needle injections do not differ in level or duration of viremia, clinical manifestations, pathology, or antibody response (*23*). Birds infected with western equine encephalitis virus or Saint Louis encephalitis virus by mosquito bite or needle exhibit no difference in viremia responses (*24*), and mosquito saliva inhibits in vitro infection of dendritic cells by dengue virus (*25*).

VEEV (family *Togaviridae*, genus *Alphavirus*) is an important emerging and reemerging pathogen of humans and equines in the neotropics. Since no effective antiviral agents or a licensed human vaccine for VEEV exists, therapy is primarily supportive and prevention relies on avoidance of mosquitoes. Outbreaks of VEE can involve hundreds of thousands of equine and human cases, spread over large regions, and can last several years (*26*).

The effect of vector feeding on vertebrate infections by VEEV has not been studied. We determined the amount of VEEV in mosquito saliva collected in vitro (*12*) but did not determine whether this amount accurately reflects transmission during blood feeding. To collect saliva in a capillary tube, we need to subject the mosquito to traumatic manipulations that may affect salivation, such as anesthetization or immobilization by removal of the legs and wings. Also, mosquitoes are usually allowed to salivate into capillary tubes for a longer time (e.g., 30 minutes) than is required for engorgement on a host. Because knowing the infectious dose transmitted by mosquitoes is important for designing vertebrate infection studies, in which needles are typically used for virus delivery, we compared the amount of VEEV transmitted by mosquitoes in vitro with that  transmitted in vivo. We also determined whether mosquito transmission affects viremia or time to death when compared with needle infections. Finally, we used tail amputations to investigate the extravascular or intravascular location of VEEV deposition during mosquito feeding.

## Methods

### Virus

We used VEEV rescued from an infectious cDNA clone derived from epidemic strain 3908 (subtype IC), a 1995 human isolate from Zulia State, Venezuela (*27*). With the exception of some IE virus strains in Mexico, subtype IC viruses are the etiologic agents of all recent VEE epidemics. Strain 3908 was passaged once in C6/36 mosquito cells before isolation of viral RNA and production of infectious cDNA. Virus recovered from baby hamster kidney cells electroporated with transcribed RNA was used for all experiments. Use of virus derived from an infectious clone minimized attenuating mutations that occur when VEEV is passaged in cell culture (*28*).

### Mosquitoes

*Ae*. *taeniorhynchus* F_1_ progeny of mosquitoes captured in Florida (*29*) were reared at 27°C and a relative humidity of 80% in a light:dark cycle of 12:12 hours. Adult females were infected intrathoracically with 4 log_10_ PFU of VEEV in a 1-μL volume 6–8 days after emergence and incubated at 27°C for 5 days with 10% sucrose provided ad libitum. Intrathoracic infection of mosquitoes with VEEV and incubation for 5 days generates saliva titers comparable to those that occur after oral infection (*12*).

### In Vivo Transmission

Thirty-nine 6- to 8-week-old National Institutes of Health (NIH) Swiss mice (Harlan, Indianapolis, IN, USA) were anesthetized with pentobarbital, and the distal portion of the tail was exposed to 1 infected mosquito. After mosquito engorgement, the tips of the tails of 29 mice were severed and immediately homogenized in 300 μL of Eagle's minimal essential medium (MEM) supplemented with 20% fetal bovine serum (FBS) in a Mixer Mill 300 (Retsch Inc., Newton, PA, USA); the tails of 10 control mice were left intact. After centrifugation at 9,000×*g* for 5 minutes, the supernatant was removed for cell culture assays and RNA extraction with a Qiagen kit (Qiagen, Valencia, CA, USA). Vero cells were injected with 30 μL of supernatant and observed for 5 days for cytopathic effects (CPEs). All CPE-positive samples were titrated by plaque assay on Vero cells.

RNA was also extracted from the pellet of the tail homogenate with Trizol (Invitrogen, Carlsbad, CA, USA). The RNA of both supernatant and tail pellet was tested for VEEV positive-strand RNA by using real-time RT-PCR with a one-step kit (Qiagen) and a Smart Cycler (Cepheid, Sunnyvale, CA, USA). Forward (5´-CATAGTCTAGTCCGCCAAGATGTT-3´) and reverse (5´-CGATAGGGCATTGGCTGCAT-3´) primers and a probe (5´-[6-FAM]CCCGTTCCAACCAATGTAT[NFQ-MGB]-3´) were used for amplification and detection, respectively. The assay consisted of reverse transcription at 50°C for 20 minutes, denaturation at 95°C for 10 minutes, and 45 cycles at 95°C for 15 seconds, 63°C for 30 seconds, and 72°C for 30 seconds. Virus titers were extrapolated from RT-PCR results by comparison with a standard curve generated from serial dilutions of a VEEV stock quantified by plaque assay.

After a mosquito probed or fed on the mouse tail, its infection was confirmed by using forced salivation into a capillary tube as described below. All 39 mice used were kept in individual cages, monitored for signs of infection, and bled retroorbitally 2 weeks later to test for seroconversion by using plaque-reduction neutralization tests.

To ensure that all virus injected into mouse tails could be recovered and detected, a series of known doses (2.9 log_10_, 2 log_10_, and 1 log_10_ PFU) was injected intradermally into the tail of a mouse. The tips were then severed and processed as described above. Each dose was tested in duplicate, and nearly all the injected virus was recovered (mean 2.7 log_10_ PFU recovered for 2.9 log_10_ inoculum, mean 1.9 log_10_ PFU recovered for 2.0 log_10_ inoculum, and mean 0.8 log_10_ PFU recovered for 1.0 log_10_ inoculum). Samples with known virus titers were also tested to ensure that freezing and thawing once did not alter virus content.

### Saliva Assays

Thirty-nine saliva samples from intrathoracically infected mosquitoes that fed on a mouse were obtained by immobilization (legs and wings removed) and forced salivation into 10-μL capillary tubes (VWR International, West Chester, PA, USA) containing immersion oil (type B, Cargille Laboratories Inc., Cedar Grove, NJ, USA). After 30–45 minutes, salivation was confirmed by appearance of bubbles at the tip of the proboscis. An additional cohort of mosquitoes was allowed to salivate for intervals (repeated in triplicate) to duplicate times of observed mosquito feeding. The oil-saliva mixture was centrifuged into 100 μL of MEM containing 20% FBS and frozen at -80°C; 30 μL was then added to Vero cells for detection of CPE. Mosquito infection was confirmed by assaying triturated bodies and legs and wings for CPE, followed by plaque assay.

### Viremia and Death

Ten 6- to 8-week-old NIH Swiss mice were infected by either 1 mosquito or intradermal injection into the ear with either 0.9 or 3.4 log_10_ PFU, which represented the range of titers injected by mosquitoes (see below). Five mice from each cohort of 10 were bled retroorbitally at 12, 24, 36, 48, 72, 96, and 120 hours postinfection, and sera were titrated by plaque assay. Mice were monitored daily until signs of encephalitis appeared, after which they were observed 4 times a day to determine time of death. The University of Texas Medical Branch Institutional Animal Care and Use Committee approved all experiments.

### Statistical Analysis

Log-transformed data were normally distributed, except for data from RT-PCR assays from mouse tail homogenate pellets on which 1 mosquito probed (this group was not compared statistically). One-way analysis of variance using Tukey's test for multiple comparisons and an unpaired *t* test were used to compare all normally distributed data with GraphPad Prism 4.0 (GraphPad Software, San Diego, CA, USA).

## Results

### In Vivo Versus In Vitro Transmission Titers

**Figure 1:**
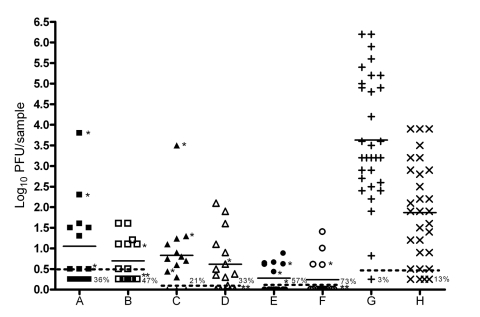
Titers of Venezuelan equine encephalitis virus (VEEV) transmitted in vitro or in vivo by *Aedes taeniorhynchus*. A, C, and E) Mosquitoes that engorged to completion (depicted by closed symbols). B, D, and F) Mosquitoes who probed but did not engorge (depicted by open symbols). The assay used to determine the virus titer was either cell culture assay (A and B, depicted by squares) or real-time reverse transcription–PCR (C and F) of tail homogenate supernatant (C and D, depicted as triangles) or pellet (E and F, depicted as circles). The last 2 cohorts (G and H) represent VEEV titers in saliva of mosquitoes allowed to salivate for 45 min (G, depicted as +) or for the same range of times (<3 min) required for mosquitoes to engorge completely on mouse tails, repeated in triplicate (H, depicted as ×). Solid horizontal lines indicate means, and horizontal dashed lines indicate detection limits for the assays. Symbols below the dashed lines indicate samples from infected mosquitoes (bodies and legs or wings positive for cytopathic effects) that were below the limit of detection for the assay, and numbers indicate the percentages for these negative samples (column A=36%, B=47%, C=21%, D=33%, E=57%, F=73%, G=3%, and H=13%). *Denotes mice that were bitten by a given mosquito that died.

To determine whether saliva collection accurately approximates the amount of VEEV transmitted during a mosquito bloodmeal, we quantified virus from saliva collected in vitro and virus deposited at sites of in vivo blood feeding. One mosquito feeding on the distal portion of a mouse tail transmitted a mean ± standard deviation of 1.1 ± 1.0 log_10_ PFU (11 PFU) as detected in the homogenate supernatant and 0.8 ± 0.9 log_10_ PFU (7 PFU) as estimated by real-time RT-PCR. These amounts were significantly lower (p<0.001) than the mean ± standard deviation amount (3.6 ± 1.5 log_10_ PFU or 4,300 PFU) deposited into capillary tubes during 30–45 minutes of salivation (Figure 1). However, the time for engorgement (<3 minutes) was much shorter than the 45 minutes allowed for in vitro salivation. Therefore, we matched times of saliva collection (range <3 minutes) to the exact engorgement times. Less VEEV (p<0.05) was still detected after in vivo transmission than after <3 minutes of in vitro salivation (1.9 ± 1.2 log_10_ PFU or 74 PFU).

The effect that the time of probing or feeding had on the titer of virus salivated was analyzed by timed saliva collections and mouse tail exposures. The amount of VEEV collected from mosquitoes that salivated in vitro for <3 minutes was significantly less than the amount collected from mosquitoes allowed to salivate for 45 minutes (p<0.0001). However, no significant difference was seen in the amount transmitted by mosquitoes allowed to completely engorge compared with mosquitoes allowed to probe only, without engorgement (p>0.05, 95% confidence interval -0.8 to 1.5 log_10_ PFU for the difference in the mean titers).

To address the possibility that some virus injected by feeding mosquitoes rapidly binds to or penetrates mouse cells and therefore is not measured by plaque assay, we also examined VEEV RNA content in mouse tails. No difference was detected between mean virus content in the mouse tail homogenate supernatants assayed by RT-PCR or plaques (Figure 1). Detection of relatively small amounts of viral RNA in tail homogenate pellets indicated that almost all virus remained in the supernatant and that infectious virus was not underestimated because of rapid penetration of cells or binding of virus to connective tissue (Figure 1).

### Location of VEEV Deposition In Vivo

To assess intravascular versus extravascular locations of VEEV deposition by mosquitoes, we amputated the distal portions of mouse tails immediately after engorgement, and mice were observed for signs of infection. Forty percent (4/10) of control mice whose tails were not amputated after mosquito feeding survived compared with 79% (23/29) of those whose tails were amputated (p = 0.04, by Fisher exact test). No mice that survived developed neutralizing antibodies. NIH Swiss mice infected with VEEV have a death rate approaching 100%, which indicates that a systemic VEEV infection did not occur in surviving animals. This suggested that nearly all saliva and associated virus were deposited extravascularly and confined to the bite site by a lack of immediate vascular dissemination. Tail amputation nearly doubled survival rates by removing this virus before replication and dissemination.

### Virus Transmitted Versus Time of Engorgement

**Figure 2:**
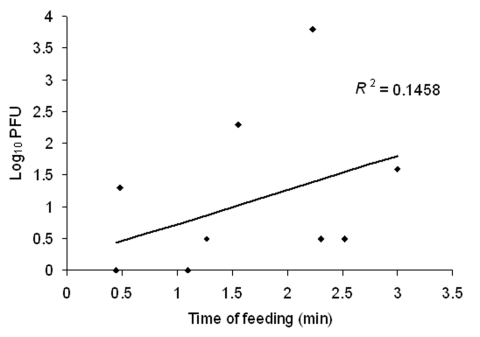
Amount of Venezuelan equine encephalitis virus transmitted into a mouse tail versus the time required for complete engorgement. Only samples from mosquitoes that completely engorged and transmitted detectable virus were included.

To assess the temporal pattern of virus deposition during blood feeding, the amount of VEEV transmitted was compared with the time required for mosquito engorgement. Figure 2 shows no correlation between feeding time and amount of VEEV in mouse tails, suggesting that most virus in saliva was deposited early during probing, with minimal deposition during engorgement.

### Effect on Pathogenesis in Mice Infected by Needle Versus Mosquito

**Figure 3:**
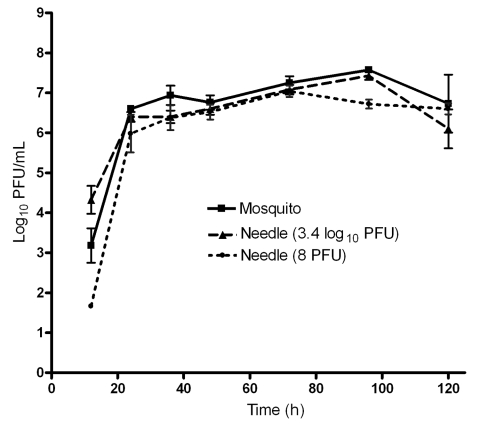
Viremia in mice infected by 1 mosquito bite or intradermally by needle injection with 2 different doses of Venezuelan equine encephalitis virus representing the range of doses delivered during blood feeding (Figure 1). Five animals per cohort were bled at each time point. Error bars indicate standard deviations.

**Figure 4:**
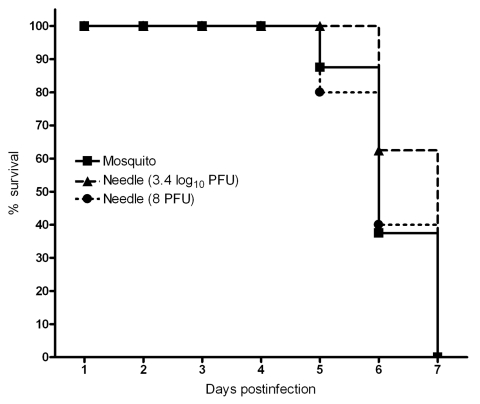
Survival of cohorts of 10 mice infected with Venezuelan equine encephalitis virus either by 1 mosquito bite or by intradermal needle injection with 2 doses representing the range of virus titers delivered during mosquito blood feeding (Figure 1).

To determine whether mosquito saliva affects pathogenesis of VEEV infection, mice were infected by either the bite of 1 mosquito or by intradermal needle injection. Two VEEV doses were used to represent the range of titers injected during blood feeding (Figure 1). Viremia from needle injection with a high dose did not differ from that generated by mosquito transmission (Figure 3). In contrast, viremia from a mosquito bite was higher than that from a needle injection of a low dose for the 12-hour (p<0.05) and 96-hour (p<0.001) time points. A significant difference in viremia (p<0.001) was also observed at the 12-hour and 96-hour time points for mice infected by needle injection of a high dose than infection of a low dose. No difference was detected in the mean survival times of mice infected by either mosquito (5.9 ± 0.6 days) or needle injection with 8 PFU (6.4 ± 0.7days) or 3.4 log_10_ PFU (6.3 ± 0.4 days) (Figure 4).

## Discussion

Studies with some (*19**–**21*) but not all (*23**,**24*) arboviruses suggest that natural infection by mosquito bite may potentiate arboviral infection compared with parenteral infection. Because the effect of mosquito transmission on infection by VEEV has not been addressed, we assessed the infectious dose delivered by a natural vector, *Ae*. *taeniorhynchus*, compared with estimated doses from saliva collections. We also evaluated the effect of mosquito VEEV transmission on pathogenesis in mice.

### In Vivo Versus In Vitro Transmission

Our results indicate that *Ae*. *taeniorhynchus* transmit less VEEV in vivo than they deposit into a capillary tube, even when saliva collection times are matched to engorgement times. We therefore caution against extended times of saliva collection in capillary tubes because mosquitoes allowed to salivate for 45 minutes expel more VEEV than those that salivate for <3 minutes, the approximate maximum time required for natural engorgement.

Our study also assessed the location of saliva deposition. As reasoned by Turell et al. (*30**,**31*), if an arbovirus were deposited intravascularly, it would quickly circulate beyond the bite site and animals with tail amputations would still become infected and die. Turell et al. reported that when the tails of suckling mice are exposed to a VEEV-infected *Ae*. *taeniorhynchus* and the tails are amputated £10 minutes later, 31%–37% survive compared with 4% of mice whose tails were not amputated (*30*). Our results indicating that the odds of dying are decreased by ≈50% for those whose tails were amputated suggest that saliva and VEEV are deposited both intravascularly and extravascularly. This conclusion is slightly different than that of Turell et al. (*30**,**31*), who concluded that mosquitoes inject most virus extravascularly and only small amounts intravascularly or that intravascular transmission occurs only occasionally. An explanation for the differences in death rates found in our studies and those of Turell et al. is that they used suckling mice, whereas we used adult mice. Two of our mice that had been only probed by an infected mosquito also became infected. Surprisingly, no VEEV was detected in the tail homogenate of these mice by either cell culture or RT-PCR. Because the 50% mouse subcutaneous lethal dose (LD_50_) for VEEV strain 3908 administered in the tail is 12 PFU (D.R. Smith, unpub. data), which is greater than the LD_50_ for injection in the thigh (*12*) and an amount detectable by our methods, virus was probably deposited primarily intravascularly in these 2 animals.

Forty percent of our mice with intact tails survived after allowing an infected mosquito to engorge. NIH Swiss mice are highly susceptible to VEEV; death rates are typically 100%. Therefore, our results and those from our previous study (*12*), which reported that infected mosquitoes often deposit <12 PFU of VEEV into capillary tubes, suggest that systemically infected *Ae*. *taeniorhynchus* frequently transmit little or no virus. In contrast to the 40% survival rate of mice with intact tails, 100% of mice infected by mosquito bite at another site died, presumably because of a difference in the site of virus deposition. The subcutaneous LD_50_ for VEEV strain 3908 administered in the tail is 12 PFU compared with <1 PFU in the leg (D.R. Smith, unpub. data). Mosquitoes may deposit different amounts of virus at different anatomic sites, depending on accessibility of blood vessels.

### Time of Engorgement and Infectious Dose Transmitted

The amount of VEEV transmitted by *Ae*. *taeniorhynchus* did not correlate with time of engorgement. However, we did not count how many times the mosquito probed before beginning to engorge. Assuming that most mosquito saliva is injected during the intradermal probing period that precedes cannulation of a blood vessel and that infection of the host correlates with the duration of salivation during probing, probing frequency could affect transmission outcome and should be investigated.

### Effects on VEE Pathogenesis of Infection by Needle Versus Mosquito

Because mosquitoes transmit a wide range of arbovirus doses, we injected mice with 2 doses that represented the range of VEEV transmitted in vivo. No difference in viremia was detected between mice infected by a mosquito than by needle injection of a high dose. However, mice infected by mosquito bite showed higher viremia titers at the early (12 hours) and late (96 hours) time points than did mice infected with a low dose given by needle. Because mice injected with the high dose also had higher viremia titers at some time points than did mice in the low-dose cohort (Figure 3), the difference in the infection by mosquito bite versus a low-dose injection by needle may indicate only that some mosquitoes transmitted doses >8 PFU. The only way to confirm this slight effect of mosquito transmission on early and late viremia would be to duplicate the exact distribution of in vivo transmission titers by needle injections. However, volumes injected by mosquitoes compared with those injected by needles would differ, as would intradermal sites of deposition. Another approach is to co-inject mosquito saliva with virus (*20**,**22*), but the same volume and site discrepancies would apply.

Although route (mosquito versus needle) or dose of VEEV had no detectable effect on death rates (Figure 4), mosquito transmission enhancement of early viremia titers could affect subsequent vector infection, and comparable studies with natural reservoir or amplification hosts are needed to assess this possibility. In preliminary studies, no difference in the viremia response of spiny rats was observed after VEEV infection by needle compared with mosquito bites (A.S. Carrara and S.C. Weaver, unpub. data).

Our results agree with reports of little or no enhancement of alphaviral infections by mosquito transmission (*24*). In several studies describing enhancement of arboviral infection by mosquito transmission, multiple mosquitoes were allowed to feed and transmit to 1 vertebrate (*19**–**21*), or salivary gland extracts from many mosquitoes were injected with virus (*20**,**22*). Because natural infection rates of mosquitoes are typically low, simultaneous transmission by >1 vector is probably rare. In addition, virus amounts injected by needle in these studies may have been less than the virus amount transmitted by mosquitoes, which would confound interpretation. Artificial conditions used for several experiments demonstrating potentiation of arbovirus infection by mosquito transmission may therefore exaggerate the true effect.

### Significance for Pathogenesis Studies

In conclusion, *Ae*. *taeniorhynchus* transmit less VEEV in vivo than they deposit in vitro into capillary tubes. Mosquito transmission of VEEV has little or no effect on the overall murine viremia profile and none on death. To design VEE pathogenesis studies that simulate natural infection, a dose range from 10 PFU to 1,000 PFU is recommended to simulate mosquitoborne infections. Because VEEV saliva titers differ among mosquito species (*12*), comparable studies should be conducted with other vectors.
